# MicroRNA-155对人肺癌95D细胞生长的影响

**DOI:** 10.3779/j.issn.1009-3419.2011.07.03

**Published:** 2011-07-20

**Authors:** 安东 秦, 涯 周, 美霞 盛, 广茹 费, 涛 任, 林 徐

**Affiliations:** 1 563003 遵义，遵义医学院免疫学教研室 Department of Immunology, Zunyi Medical College, Zunyi 563003, China; 2 563003 遵义，遵义医学院医学物理学教研室 Department of Medical Physics, Zunyi Medical College, Zunyi 563003, China; 3 200120 上海，同济大学附属上海东方医院呼吸内科 Department of Respiratory Medicine, Shanghai East Hospital, Tongji University, Shanghai 200120, China

**Keywords:** MicroRNA-155, 肺肿瘤, 细胞增殖, 细胞周期阻滞, SOS1, MicroRNA-155, Lung neoplasms, Cell proliferation, Cell cycle, SOS1

## Abstract

**背景与目的:**

近年来的研究发现miRNAs（microRNAs）家族成员miR-155与肺癌的发生相关，然而具体机制不明。本研究拟探讨上调miR-155表达对人肺癌95D细胞体外生长的影响及其可能的作用机制，为后续深入研究miR-155在肺癌发生、发展中的作用提供新的实验依据。

**方法:**

人肺癌95D细胞分为空白对照组、miR-155阴性对照组和miR-155转染组，分别作加入转染试剂、转染miR-155阴性对照和转染miR-155处理。Real-time PCR特异性探针法检测转染后95D细胞中miR-155的表达水平；应用MTT法检测miR-155对95D细胞增殖的抑制作用；流式细胞术检测95D细胞周期的分布；Western blot检测95D细胞SOS1蛋白的表达变化。

**结果:**

Real-time PCR结果显示，与对照组相比，转染组95D细胞miR-155的表达水平明显增加（*P* < 0.05）；镜下可见转染miR-155的95D细胞生长减缓，形态发生改变；MT结果表明miR-155转染后，95D细胞增殖受到抑制，转染组和对照组存在明显差异（*P* < 0.05）；流式细胞术分析显示，与对照组相比，转染miR-155的95D细胞周期发生改变，G_0_/G_1_期细胞明显增多，而S期则明显减少（*P* < 0.05）；Western blot结果显示转染组95D细胞SOS1蛋白的表达明显降低（*P* < 0.05）。

**结论:**

miR-155能明显抑制人肺癌95D细胞的体外生长，这可能与miR-155下调SOS1表达及诱导95D细胞G_0_/G_1_期阻滞相关。

## Abstract (en)

**Background and objective:**

Recent studies suggest that miR-155 is involved in lung tumorgenesis, whereas the precise mechanism has not yet been characterized. The aim of this study is to investigate the effects of overexpression of miR-155 on the growth of human lung cancer 95D cells *in vitro* and its possible mechanism, and thus to provide experimental evidence for further researching on the role of miR-155 in the pathogenesis and development of lung cancer.

**Methods:**

miR-155 mimics control and miR-155 mimics were tranfected into human lung cancer 95D cells by FuGENE^®^ HD Transfection Reagent respectively *in vitro*. The relative expression level of miR-155 in 95D cells was determined using specifc probe of real-time PCR afer transfection. The proliferation of 95D cells was detected by MT assay. The cell cycle was analyzed by flow cytometry. The expression of SOS1 protein was measured by Western blot.

**Results:**

Compared with control groups, the expression level of miR-155 was signifcantly increased in miR-155 mimics transfected group (*P* < 0.05). The proliferation of miR-155-transfected 95D cells was signifcantly inhibited (*P* < 0.05). The percentage of G_0_/G_1_ phase cells was increased signifcantly in miR-155-transfected 95D cells, while the percentage of S phase was remarkably reduced (*P* < 0.05). Furthermore, the expression of SOS1 in miR-155-transfected 95D cells was signifcantly down-regulated (*P* < 0.05).

**Conclusion:**

miR-155 could signifcantly inhibit the growth of human lung cancer 95D cells *in vitro*, which might be closely related to miR-155 induced G_0_/G_1_ phase arrest.

MicroRNAs（miRNAs）是一组内源性、长约22 nt的非编码小RNA，其主要在转录后水平通过与靶mRNA的3´非翻译区（3′UTR）结合，导致目的mRNA的降解或蛋白质翻译抑制，而负调控目的基因的表达^[1]^。miRNAs参与细胞的分化、增殖、凋亡等多种生物学进程。近年来的研究^[2, 3]^表明，miRNAs的突变和表达异常与肿瘤的发生、发展密切相关，可发挥癌基因和抑癌基因的作用，促进或抑制肿瘤的生长、侵袭和转移。

肺癌是最常见的恶性肿瘤，高居恶性肿瘤死亡率的首位^[4]^。近年来，大量的研究^[5-8]^显示，miRNAs与肺癌的生长、侵袭和转移、预后相关。miR-155是miRNAs家族中的重要成员，有研究^[8-10]^报道，与正常肺组织相比，miR-155在肺癌组织中表达异常，与肺癌的预后相关。但目前关于miR-155与肺癌具体关系的研究还比较少。我们通过观察体外转染miR-155对人肺癌95D细胞体外生长的影响，探讨其可能的作用机制，为进一步深入研究miR-155与肺癌的关系提供前期理论基础和实验依据。

## 1 材料与方法

### 1.1 细胞株

人源性肺巨细胞癌细胞株95D，购自中国科学院上海生化所。

### 1.2 主要试剂

RPMI-1640培养基（Hyclone）；优质胎牛血清FBS（Solarbio）；hsa-miR-155 mimics、hsa-miR-155 mimics negative control（上海生工生物工程有限公司合成）；FuGENE® HD Transfection Reagent（Roche）；Trizol试剂（Invitrogen, USA）；RevertAid^TM^ First Strand cDNA Synthesis Kit（Fermentas）；TaqMan^®^ MicroRNA Assay Kit（Ambion）；MTT试剂盒（Sigma）；兔抗人SOS1单克隆抗体、HRP标记羊抗兔二抗（Cell Signaling Technology）；PI（propidium iodide）（Abcam）。

### 1.3 细胞培养和转染

人肺癌95D细胞在含10%FBS（胎牛血清）、100 U/mL青霉素、100 mg/mL链霉素、1 mmol/L L-谷氨酰胺的RPMI-1640的培养基中，37 ℃、5%CO_2_全湿度培养。细胞转染实验分组：①空白对照组（Mock）：仅加入FuGENE® HD Transfection Reagent转染试剂；②阴性对照组（miR-155 cont）：转染miR-155 mimics negative control；③ miR-155转染组（miR-155）：转染miR-155 mimics（40 pM, 80 pM, 160 pM）。转染方法按照FuGENE®HD Transfection Reagent说明书操作。

### 1.4 RT-PCR检测miR-155的表达

收集上述转染48 h后的95D细胞，Trizol一步法提取总RNA，用miR-155发夹状引物按试剂盒说明书操作逆转录合成cDNA；应用LightCycler（Roche Molecular Biochemicals, Mannheim, Germany）定量PCR仪对miR-155成熟体进行定量检测。miR-155成熟体TaqMan探针由TaqMan^®^ MicroRNA Assay Kit提供，PCR反应条件按试剂盒说明书操作。在扩增结束后将每对引物的扩增产物进行2%凝胶电泳分析，以确定扩增产物的特异性和灵敏度。miR-155成熟体相对表达水平计算：特异基因的DNA含量/内参GAPDH的含量=目的基因的相对表达水平。

### 1.5 MTT法检测miR-155对95D细胞的增殖抑制

收集上述各实验组细胞制备成单细胞悬液，调整浓度为（4-5）×10^5^/mL接种于96孔培养板（200 μL/孔），每组细胞设10个平行孔。37 ℃、5%CO_2_培养72 h，每孔加入5 mg/mL的MTT 20 μL，继续孵育4 h，弃孔内培养液，每孔加入150 μL二甲基亚砜（DMSO），振荡10 min，待结晶物充分溶解后，酶标仪检测570 nm处各孔OD值。

### 1.6 流式细胞术分析细胞周期分布

离心收集各组95D细胞，弃上清，用预冷PBS洗涤细胞两次，加入预冷70%乙醇，于4 ℃固定过夜，PBS洗涤收集细胞，PBS重悬（1×10^6^个/mL），吸取100 μL细胞悬液，加RNase A（100 μg/mL）和PI（50 μg/mL），4 ℃避光孵育30 min，PBS洗涤重悬后用BD FACSCalibur流式细胞仪检测细胞周期的分布情况。

### 1.7 Western blot检测SOS1蛋白的表达

收集各组细胞，加入细胞裂解液裂解细胞，提取总蛋白，Bradford法蛋白定量，进行SDS-PAGE电泳，将电泳分离的蛋白电转移至PVDF膜上（14 V、80 min），5%脱脂牛奶封闭，加兔抗人SOS1单克隆抗体（1:200稀释），4 ℃过夜，TBST洗3次，每次10 min。加入HRP标记的羊抗兔二抗（1:2, 000稀释），37 ℃，孵育1 h，TBST缓冲液充分洗膜3次，每次10 min，再用TBS洗膜1次，10 min，发光压片显色，以β-actin作为内参，分析结果。

### 1.8 统计学方法

采用SPSS 16.0进行统计学分析，多组间比较采用单因素方差分析，以*P* < 0.05为有统计学差异。

## 2 结果

### 2.1 miR-155在95D细胞内的表达水平

为了检测miR-155的转染效率，转染后48 h用实时荧光定量PCR对miR-155成熟体的表达进行检测，结果显示，与Mo c k组及miR-155 cont组比较，miR-155转染组95D细胞miR-155的表达明显增加（*P* < 0.05）（图 1），结果表明miR-155转染效率良好。

**1:**
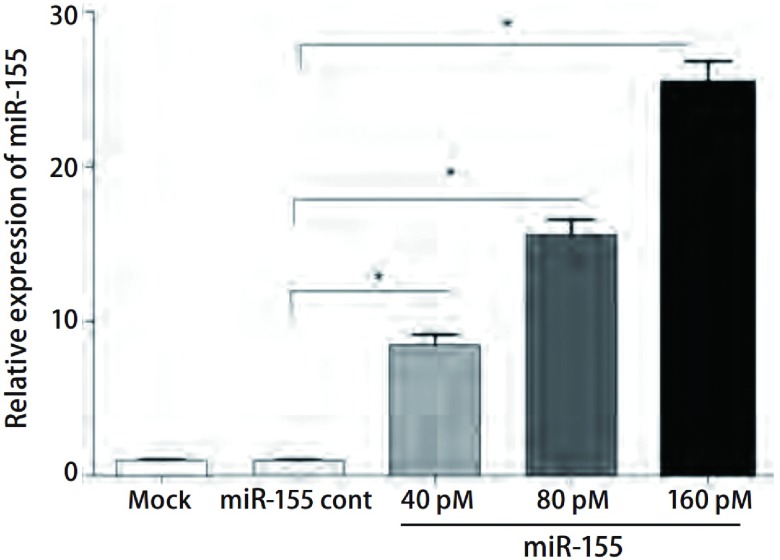
RT-PCR法检测转染后人肺癌95D细胞miR-155的相对表达水平 Relative miR-155 expression in human lung cancer 95D cells transfected with miR-155 mimics detected by RT-PCR

### 2.2 miR-155转染对95D细胞体外生长的影响

miR-155转染48 h后，光学显微镜下观察发现，Mock组和miR-155 cont组细胞生长状态良好，与Mock和miR-155 cont组相比，miR-155转染组95D细胞的生长发生明显变化：数目减少，细胞皱缩变圆，贴壁不牢，部分脱落（图 2A），且具有剂量依赖性，且结果显示miR-155转染剂量为80 pM和160 pM时，两者对95D细胞生长的抑制作用无明显差异，在后续的研究中均采用80 pM的剂量进行实验。MTT结果也表明，与对照组相比，转染miR-155能明显抑制95D细胞的增殖（*P* < 0.05）（图 2B）。

**2:**
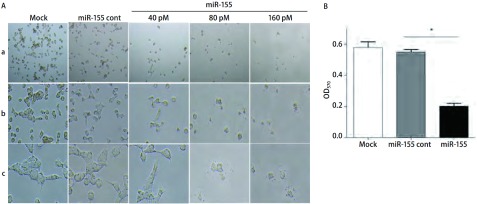
miR-155转染对人肺癌细胞95D体外生长的影响。A：光学显微镜图（a: ×100; b: ×200; c: ×400）；B：MTT法检测miR-155对人肺癌95D细胞增殖的抑制作用。 The effects of miR-155 transfection on the growth of human lung cancer 95D cells *in vitro*. A: Observed under light microscope (a: ×100; b: ×200; c: ×400); B: Detected by MTT assay.

### 2.3 miR-155转染对95D细胞周期的影响

FACS检测转染组及对照组细胞周期的分布，结果显示转染miR-155的95D细胞G_0_/G_1_期细胞比例明显增加，而S期比例则明显减少，与Mock组和miR-155 cont组比较具有统计学意义（*P* < 0.05）（图 3），结果表明上调miR-155的表达可以诱导95D细胞的G_0_/G_1_期阻滞。

**3:**
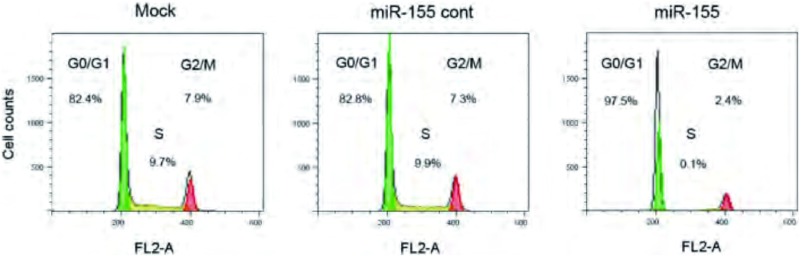
miR-155转染影响人肺癌95D细胞周期 Effect of miR-155 over-expression on cell cyle profile in human lung cancer 95D cells

### 2.4 miR-155转染对人肺癌95D细胞中SOS1蛋白表达的影响

Western blot分析显示，与两个对照组比较，miR-155可以明显抑制95D细胞中SOS1蛋白的表达（*P* < 0.05）（图 4）。

**4:**
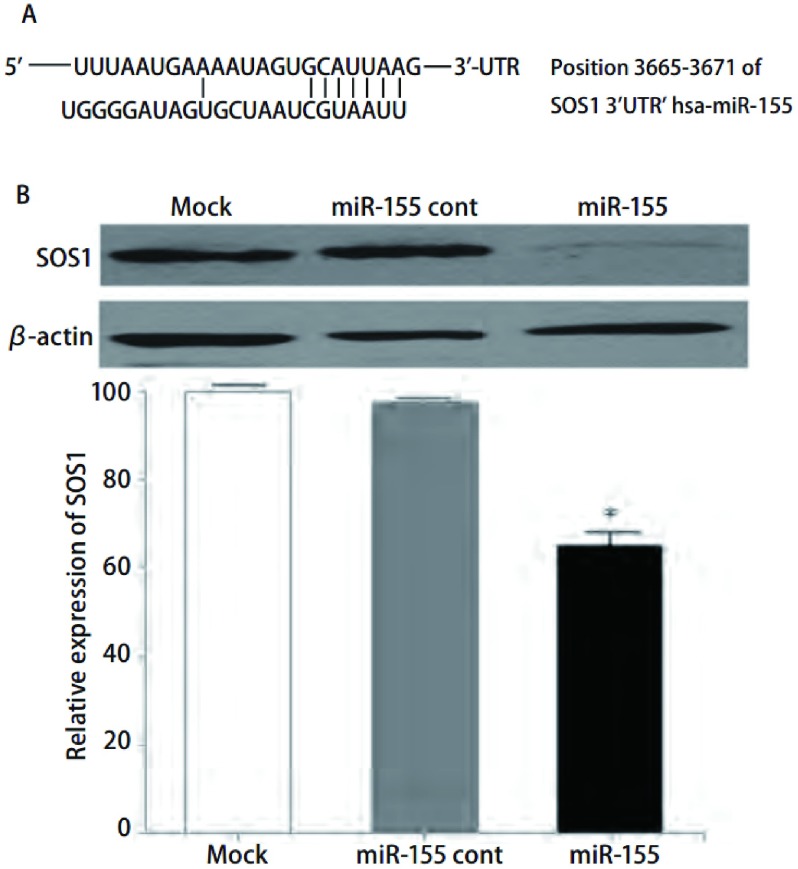
miR-155转染对人肺癌95D细胞SOS1表达的影响。A：TargetScan5.1软件分析SOS1的3′UTR区域的hsa-miR-155的互补结合位点；B：miR-155转染组与其它两对照组的SOS1蛋白表达的Western blot印迹图。 Effect of miR-155 transfection on the expression of SOS1 in human lung cancer 95D cells. A: Complementarity site for miR-155 in the 3′ UTR region of SOS1; B: The expression of SOS1 protein in human lung cancer 95D cell by Western blot.

## 3 讨论

近年来，miRNAs与肺癌的发生、发展的关系已成为生命科学研究的热点。与肺癌相关的多种miRNAs相继被发现，Hayashita等^[11]^首先在肺癌中检测出miR-17-92簇的过表达，特别是小细胞肺癌，这提示它很可能具有癌基因功能。随后的研究^[12]^显示，*miR-17-92*基因簇的成员miR-17-5p可通过抑制其靶基因Rb2的表达，从而促进肺上皮祖细胞的高度增殖和低分化，导致细胞的恶性变和肺癌的发生。Zhang等^[13]^研究表明，miR-21在非小细胞肺癌（non-small cell lung cancer, NSCLC）中明显上调，通过与抑癌基因*PTEN*的3′UTR端结合，下调*PTEN*的表达，阻止其翻译，促进肿瘤细胞的增殖。此外，Sun等^[14]^通过构建表达miR-126的质粒，转染到NSCLC A549细胞系，发现无论在体内或体外miR-126的过表达均可下调EGFL7的表达，从而抑制肿瘤细胞的生长。这些研究表明，miRNAs是肺癌发生、发展的重要调控分子，具有癌基因或抑癌基因的作用。

miR-155位于BIC基因的第3个外显子内，其表达水平受到*BIC*基因表达和miRNAs本身加工的调控^[15]^，miR-155是一种多功能的miRNA，其表达水平的变化与多种肿瘤密切相关。在血液系统肿瘤中，miR-155在多种B细胞淋巴瘤中过表达，在弥漫性大B细胞淋巴瘤中表达水平最高^[16, 17]^。Jiang等^[18]^最近的研究表明miR-155通过负调控肿瘤抑制基因*socs1*，促进乳腺癌细胞的增殖，表现为癌基因的作用。目前，miR-155在肺癌发生、发展过程中的确切功能和机制仍不清楚。我们在研究中采用体外转染技术将miR-155转染人肺癌95D细胞，MTT法检测发现miR-155能明显抑制人肺癌95D细胞的体外增殖。而新近的研究^[19, 20]^结果表明miR-155与NSCLC患者预后的相关性可能因肺癌的病理学类型而不同，在腺癌（adenocarcinomas, ACs）患者中，miR-155表达升高，预后较差；而对鳞癌（squamous cell carcinomas, SCCs）且伴淋巴结转移的患者分析表明，miR-155表达升高，患者的5年生存率却明显增加^[20]^。此外，Volinia等^[21]^发现miR-155在胰腺癌中表达下调。Dorsett等^[22]^研究显示miR-155能通过与*Aicda*的3′UTR结合，下调AID（activation-induced cytidine deaminase）的表达及依赖AID的Myc-Igh易位发生的频率，从而降低细胞癌变的风险。这些研究表明，miRNAs发挥癌基因或抑癌基因的功能可能因肿瘤的类型和病理学亚型不同而表现出功能的差异。FCM进一步分析显示，miR-155能诱导人肺癌95D细胞G_0_/G_1_期阻滞。此前的多项研究也显示miRNAs能调控肺癌细胞的细胞周期，Bandi等^[23]^发现上调miR-15a和miR-16的表达能将NSCLC细胞周期阻滞于G_0_/G_1_期。另一项研究^[24]^显示，miR-107和miR-185可使肺癌H1299细胞和A549细胞停滞在G_1_期。

为了进一步探讨miR-155对人肺癌95D细胞体外生长的抑制机制，我们采用TargetScan5.1筛选发现，SOS1的3′UTR具有与miR-155互补的序列，*SOS1*可能是miR-155的靶基因，*SOS1*是许多生长因子受体和粘附因子受体信号传导的下游分子，是Ras信号途径的一个重要分子，其活化能促进细胞的增殖和粘附。Timofeeva等^[25]^发现SOS1在前列腺癌中水平升高，下调前列腺癌细胞PC3和DU145中SOS1的表达，细胞的增殖、迁移及侵袭能力明显减弱。我们通过Western blot检测转染miR-155的人肺癌95D细胞SOS1表达水平，结果显示转染miR-155能明显下调人肺癌95D细胞SOS1表达，通过研究分析，我们推测miR-155很可能通过下调SOS1的表达、诱导细胞周期阻滞对人肺癌95D细胞的体外生长发挥抑制作用，但是，miR-155对于肺癌生长的作用和机制有待进一步的研究。

总之，我们的研究表明miR-155可以明显抑制人肺癌95D细胞的体外生长，这可能与与其抑制SOS1的表达和诱导细胞周期阻滞有关，为深入研究miR-155在肺癌细胞生长中的作用提供了前期的实验依据，同时可为临床肺癌生物治疗提供新的靶点和方向。
